# Oral Plaque from Type 2 Diabetic Patients Reduces the Clonogenic Capacity of Dental Pulp-Derived Mesenchymal Stem Cells

**DOI:** 10.1155/2019/1516746

**Published:** 2019-01-14

**Authors:** Antonella Bordin, Francesca Pagano, Eleonora Scaccia, Matteo Saccucci, Iole Vozza, Noemi Incerti, Antonella Polimeni, Elena Cavarretta, Isotta Chimenti, Elena De Falco

**Affiliations:** ^1^Department of Medical Surgical Sciences and Biotechnologies, Sapienza University of Rome, C.so della Repubblica 79, 04100 Latina, Italy; ^2^Department of Oral and Maxillo-Facial Sciences, Sapienza University of Rome, Rome, Italy

## Abstract

Type 2 diabetes (T2D) is a major metabolic disease and a key epigenetic risk factor for the development of additional clinical complications. Among them, periodontitis (PD), a severe inflammatory disease ascribable to a dysregulated physiology and composition of the oral microbiota, represents one of the most relevant complications. Periodontitis can impact the structure of the tooth and likely the stem and progenitor cell pool, which actively contributes to the periodontal microenvironment and homeostasis. Modifications of the oral plaque play a key role in the etiopathogenesis of PD caused by T2D. However, to what extent the biology of the progenitor pool is affected has still to be elucidated. In this short report, we aimed to explore the biological effects of oral plaque derived from T2D patients with PD in comparison to non-diabetic patients with PD. Oral plaque samples were isolated from T2D and non-diabetic subjects with PD. Dental pulp stem cells (DPSCs), derived from the premolar tooth, were conditioned for 21 days with oral plaque samples and tested for their clonogenic ability. Cultures were also induced to differentiate towards the osteogenic lineage, and ALP and osteocalcin gene expression levels were evaluated by real-time qPCR. Results have shown that the number of clones generated by DPSCs exposed to T2D oral plaque was significantly lower compared to controls (ctl). The multivariate analysis confirmed that the decreased clonogenesis was significantly correlated only with T2D diagnosis. Moreover, the effect of T2D oral plaque was specific to DPSCs. Indicators of osteogenic differentiation were not significantly affected. This study provides a new biological insight into the effects ascribable to T2D in PD.

## 1. Introduction

Type 2 diabetes (T2D) is a very common metabolic disease caused by resistance to insulin and consequent systemic hyperglycaemia. The pathological effects induced by T2D are chronic and exhibit multifaceted features, as they imply profound alterations in both the endocrine asset and metabolic/physiological functions of several biological systems [1–3], including the oral microenvironment [4, 5]. In this regard, periodontitis (PD), a severe inflammatory disorder of the periodontium caused by oral bacterial challenge, is a complication of T2D [6]. In fact, patients affected by T2D are more susceptible to PD. The periodontium is a highly vascularised system, and oral plaque possesses intrinsic biological properties such as the pivotal chemotactic ability for immune cells [7]. Type 2 diabetes severely impairs the architecture of periodontal tissue and interferes with basic cellular functions such as phagocytosis, migration, and more importantly the innate immune response provided by the epithelial mucosa of the periodontium. Importantly, the unbalanced inflammatory and dysmetabolic state in T2D significantly affects the composition of the bacterial film in the oral plaque, therefore impairing periodontal integrity and function [8]. This scenario is further exacerbated by modifications both in macro and microcirculation of the periodontium, ascribable to T2D due to the increase in glycation end-products (AGEs), oxidative stress and deregulation of the endothelial barrier [8]. Intriguingly, PD and diabetes mutually influence each other in a bidirectional fashion: diabetes increases proinflammatory levels of soluble mediators, therefore reinforcing periodontal inflammation; in turn, PD influences the glycaemic index in diabetic patients [9]. Moreover, epidemiological studies have demonstrated that PD is strictly associated with increased cardiovascular risk as a direct consequence of endothelial dysfunction following changes of oral plaque [10]. Notably, T2D-induced oral plaque modifications are not limited to the sole periodontium, but they could likely affect defined cell populations of dental origin and mainly progenitors of mesenchymal origin, which reside in the tooth canal opening in the periodontium. Dental pulp stem cells (DPSCs) have been defined as adult progenitor cells of ectodermal origin, exhibiting mesenchymal features [11–13], including clonogenic and self-renewal capacity, mesodermal-like differentiation, and mainly osteogenic, mineralization, and regeneration capabilities [14, 15]. Interestingly, transplanted DPSCs in streptozotocin-induced diabetic mouse models have confirmed the ability of DPSCs both to enhance glucose tolerance in a paracrine manner [16, 17] and to restore vascular function [18, 19]. Dental pulp stem cells have been also reported as effective candidates to generate insulin-producing cells [20–22], parallel to a peculiar and attractive feature of anti-inflammatory and nociceptive soluble mediator-based secretion [21]. Importantly, the progression of PD in the presence of T2D is certainly dependent on the plaque-host relationship, which may vary between individuals. To date, biological and molecular mechanisms underlying the interplay between DPSC properties and alterations of the oral microenvironment caused by T2D are still to be addressed. Here, we investigate the effects of T2D-related oral dysbiosis and its potential biological consequences that might involve DPSCs, responsible for the dental stemness homeostasis and regenerative properties. Accordingly, we hypothesize a direct biological effect of T2D oral plaque on DPSC function and differentiation beyond the sole involvement of the periodontium. We found that oral plaque obtained from T2D patients with PD significantly reduced the clonogenic ability of DPSCs compared to that obtained from nondiabetic patients with PD, without impacting osteogenic differentiation, analysed by the expression of both alkaline phosphatase (ALP) and osteocalcin, known to represent specific markers of the osteoblast lineage [23] and to be involved in the mineralization of the extracellular matrix and therefore in osteogenesis [24]. Notably, the decrease in clonogenic potential of DPSCs was independent of the main specific clinical features of diabetic patients, such as age and sex.

## 2. Materials and Methods

### 2.1. Isolation, Culture, and Characterization of Dental Pulp Stem Cells (DPSCs) by Flow Cytometry

Dental pulp stem cells (DPSCs) were obtained from explants of dental pulp from one subject undergoing tooth extraction for orthodontic treatment and according to previous reports [15, 25]. Briefly, the premolar tooth was kept in a physiological solution after extraction and then in milk. Afterwards, the pulp was collected, chopped in a petri dish containing PBS and 1% penicillin/streptomycin, and left to adhere for 2 hours at 37°C in 5% CO_2_ in the incubator. Afterwards, PBS was removed and replaced by complete media composed of DMEM-low glucose supplemented with 20% FBS and 1% penicillin/streptomycin (all Gibco). After 72 hours, nonadherent cells were removed and fresh complete media were added. DPSCs were expanded up to the third passage by trypsinization and subcultured at 4000 cells/cm^2^ in complete media. Cell growth and viability were monitored by trypan blue exclusion assay. Cultures were characterized by flow cytometry analysis. At passage 3, DPSCs were trypsinized and resuspended in FACS buffer (PBS/2% foetal bovine serum) for immunophenotype assessment. Cells were stained with indirectly or directly conjugated primary antibodies against CD44, CD105, CD117, CD90 (all Abcam, Cat. N. ab44967, ab6124, ab23894, and ab5506), CD34 (Miltenyi Biotec, Calderara di Reno, Bologna, Italy, Cat. N. 130-081-001), and CD73 (BioLegend, San Diego, CA, USA, Cat. 344005). Cells were incubated with primary antibodies for 30 minutes, followed by staining with secondary antibodies (only if indirectly conjugated) [11–13, 26]. Data was acquired by cytofluorimeter (FACSAria II, BD, San Jose, CA, USA) and analysed by Diva Software (v6.1.1, BD, San Jose, CA, USA). This study was approved by the Ethical Committee of Policlinico Umberto I, Rome, Italy (protocol number 4336, 02/02/2017). Experiments were conducted in accordance with the 1964 Helsinki Declaration regarding the study involving human participants. Informed consent was obtained from all subjects before the surgical procedure. Subjects were all deidentified by employing a code (from B-F (T2D) and from G-I (ctl)). The clinical features of all patients are reported in Table 1. Subcutaneous adipose stromal cells (ASCs, primary cell lines 55P and 56P) were isolated as previously described [11, 27], according to the approved protocol (Ethical Committee of “Sant'Andrea” Hospital in Rome, Ref. 49_2013/28.01.2016).

### 2.2. Oral Plaque Isolation

Oral plaque was isolated as previously reported [7]. Briefly, single plaque samples were firstly spun at 400*g* for 5 minutes at 4°C in order to concentrate the plaque. Afterwards, the pellet was weighed for the following data normalization and mechanically homogenised using a pestle in ice. The plaque was then diluted in 1 ml of sterile PBS and centrifuged at 4°C and 600*g* for 15 minutes. The supernatant was then collected and filtered by using a 0.45 *μ*m mesh. Oral plaque suspensions were freshly used.

### 2.3. Clonogenic and Osteogenic Differentiation Assay

Cultures were conditioned with oral plaque from diabetic and ctl patients, by seeding DPSCs at a low density (10 cells/cm^2^) in complete media, as previously described [11–13, 26], then supplemented with an equal amount of oral plaque-derived suspensions (volume ratio 1 : 1) for 21 days. Fresh oral plaque suspensions were replaced every 3 days. At the end of the incubation time, cells were fixed with 4% paraformaldehyde for 1 hour at room temperature and then stained with Giemsa (Sigma) in order to identify nuclear and/or cytoplasmic morphology of colony-forming units (CFU). The number of clones was quantified by an optical microscope and then normalized to the amount (mg) of the oral plaque seeded [11–13, 26]. For osteogenic differentiation tests, DPSCs were cultured for 21 days in a StemPro® Osteogenesis Differentiation Kit (5 × 10^3^ cells/cm^2^, Gibco, Cat. N. A10072-01) [12, 13] supplemented with an equal amount of oral plaque-derived suspensions (volume ratio 1 : 1). Afterwards, calcium deposition was analysed incubating the cells for 1 hour at room temperature with a 2% Alizarin red solution at pH 4.1-4.3 (Sigma, St. Louis, USA, Cat. N. A5533) [13, 26].

### 2.4. Real-Time PCR

Total RNA was isolated (RNeasy kit, Qiagen), and c-DNA was obtained and amplified by the SensiMix SYBR Hi-ROX Kit (Bioline, London, UK). Templates were amplified by the 7900HT Fast Real-Time PCR System (Applied Biosystems, Cheshire, UK) for 40 cycles according to the following protocol: 95°C for 15 seconds, 56-58°C for 15 seconds, and 72°C for 15 seconds. Primer sequences used were the following: forward—GATGTGGAGTATGAGAGTGAC and reverse—GGTCAAGGGTCAGGAGTTC (ALP), forward—TGAAAGCCGATGTGGTCAG and reverse—CAGCGAGGTAGTGAAGAGAC (osteocalcin), and forward—ACAGTCAGCCGCATCTTC and reverse—GCCCAATACGACCAAATCC (GAPDH). GAPDH was considered a housekeeping gene. The reaction products were analysed by SDS 2.1.1 software (Applied Biosystems, Cheshire, UK).

### 2.5. Statistical Analysis

Statistical analysis was performed by using GraphPad Prism 5 software (San Diego, USA). Comparison between two groups was performed by *t*-test. Multivariable linear regression analyses were conducted to adjust for potential confounders, and computation was performed with SPSS 20 (IBM). A *p* value < 0.05 has been considered statistically significant. Data are presented as mean ± standard error, unless specified.

## 3. Results

**Figure 1:**
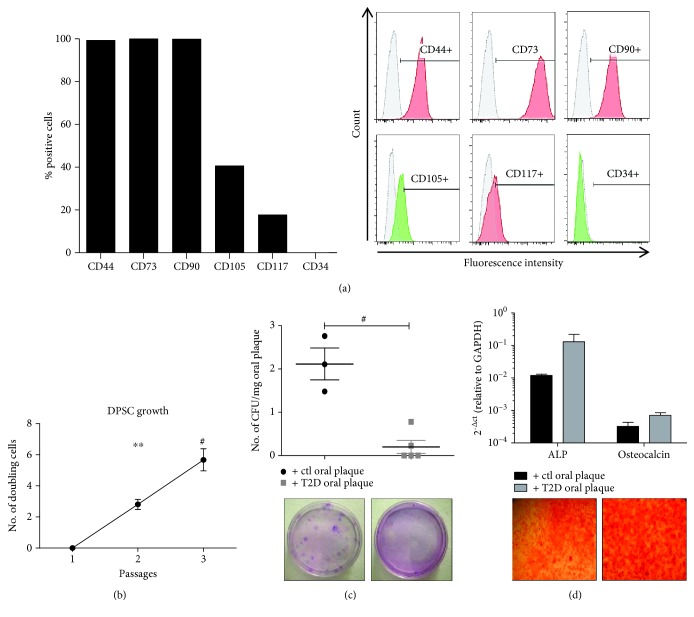
(a) Flow cytometry analysis of DPSCs after isolation. The graph shows that DPSC cultures exhibit a mesenchymal-like immunophenotype with positivity to the main stromal markers and negativity to the hematopoietic marker CD34. Representative cytofluorimetric histograms are displayed on the right (red and green histograms and APC and FITC fluorochromes, respectively). (b) Cell growth profile of DPSCs. The graph displays that the number of doubling cells is significantly increased at higher passages. Results were normalized to passage 1. ^∗∗^*p* < 0.01, ^#^*p* < 0.0001. (c) The graph shows that the number of clones generated by DPSCs in the presence of T2D oral plaque is significantly lower compared to that in controls (ctls). The number of CFU was normalized to the amount of oral plaque (mg). Below the graph, representative optical images of CFU stained with Giemsa are shown. ^#^*p* < 0.0001. (d) Osteogenic differentiation of DPSCs. The graph displays the gene expression levels of ALP and osteocalcin which are unaltered between the two treatments. Below the graph, representative optical images of osteogenic differentiation of DPSCs stained with Alizarin red. Magnification: 10x.

In order to test the influence of T2D patient-derived oral plaque on DPSC biology in the presence of PD, we have firstly isolated and characterized the primary cell line of DPSCs from the premolar tooth. Flow cytometry analysis showed that DPSCs exhibited a mesenchymal stem cell-like immunophenotype [11–13, 26] with high expression of stromal markers such as CD44, CD90 and CD73 and a moderate and low positivity for CD105 and CD117, respectively (Figure 1(a)). The expression of CD34 (hematopoietic marker) was undetectable. The DPSC cultures were expanded up to the third passage, when they exhibited the highest number of doubling viable cells (Figure 1(b), *p* < 0.0001 and *p* < 0.01, passage 3 vs. passages 1 and 2, respectively). In order to evaluate their clonogenic ability, cells were then cultured at a low density for 21 days by supplementing the culture media with oral plaque preparations obtained from T2D patients (*N* = 5) with PD. Cultures supplemented with oral plaque from non-diabetic individuals with PD were used as ctl (*N* = 3). The clinical features of subjects enrolled in this study are displayed in Table 1. Smoking, surgical interventions, and infections have been considered exclusion criteria. Results showed a striking and significant decrease in the number of clones generated by DPSCs when conditioned with oral plaque derived from T2D patients with PD compared to ctl (Figure 1(c), *p* < 0.0001). Since the average age of donors in the two groups appeared to differ, we performed a multivariable linear regression analysis to adjust for this potential confounder. The multivariable regression analysis indicated a significant association between the presence of T2D and decreased clonogenesis independently of the age of the patient (Table 2). Afterwards, we induced DPSC differentiation into the mesodermal lineage, by priming the cultures for 21 days towards the osteogenic phenotype in the presence of both types of oral plaque, and we quantified the expression of the main osteogenic genes including ALP and osteocalcin [28]. Results showed that T2D oral plaque did not significantly affect the osteogenic differentiation, although a trend of ALP to increase in cultures supplemented with oral plaque samples derived from T2D patients with respect to ctl was observed (Figure 1(d)).

**Figure 2:**
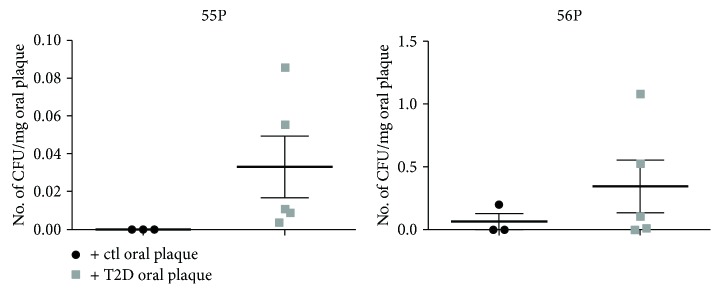
The graphs display the number of clones (CFU) generated from two distinct primary lines (55P and 56P) derived from subcutaneous adipose tissue and stimulated with ctl and T2D oral plaque, highlighting that there is no significant difference between the treatments. The number of CFU was normalized to the amount of oral plaque (mg).

Finally, in order to verify whether the effect of T2D oral plaque was specific to DPSCs, we tested the samples on two distinct human primary subcutaneous ASC lines (55P and 56P). Results have shown that the number of CFU was not significantly affected by T2D oral plaque (Figure 2), therefore suggesting a specific effect mainly related to the dental microenvironment.

## 4. Discussion

In this study, we have shown that oral plaque derived from T2D patients with PD impacts negatively on the clonogenic ability of DPSCs, highlighting a potential biological mechanism by which diabetes may contribute to periodontal damage. To the best of our knowledge, the direct effect of T2D-derived plaque on DPSCs has never been addressed. Our set of diabetic patients has developed PD, whose etiopathogenesis is linked to a wide range of alterations triggered in the mouth. These include changes of the periodontal microbiota with respect to healthy individuals [28], associated in particular with the increase in defined pathogens [29–31], known to antagonize the immune response in patients with PD [32]. Although we did not identify the nature of the biofilm in our oral plaque samples, it is plausible that the microbiological profile has been profoundly modified in diabetic patients [8, 33, 34]. Notably, the number of CFU generated by DPSCs was negatively affected by oral plaque from this set of T2D patients, despite the absence of significant statistical correlations with clinical parameters and potential interpatient variability, known to play a role in the progression of PD even in relation to the oral microenvironment [28]. Intriguingly, when subcutaneous ASCs (stromal cells of different tissue origin from DPSCs) are similarly treated with T2D oral plaque, the effect is not reproducible in our conditions, indicating that the ability of T2D to decrease the clonogenic efficiency is specific to DPSCs and likely restricted to the dental microenvironment.

The low number of subjects enrolled in this study certainly represents a limitation. Nevertheless, we cannot rule out that the alterations of the oral plaque mediated by diabetes with regard to the intrinsic properties of DPSCs represent a priming and sufficient event able to transversely affect individuals at any age and gender, therefore highlighting non-cumulative effects over the time. Additionally, we have detected a trend towards increased osteogenic differentiation in the presence of T2D oral plaque (although not statistically significant). This result is only apparently discrepant from the majority of the studies in literature, showing that diabetes severely compromises osteogenic differentiation [28, 35, 36]. However, our result should be interpreted in the light of the clonogenesis result. In fact, normally, the biological events related to the clonogenic and differentiation properties are mutually exclusive and strictly dependent on the tissue of origin [11, 23]. In the majority of adult progenitor populations, including DPSCs, the balance between these two processes is finely tuned, and pathological insults as T2D are able to deregulate both phases. Thus, the modifications of the oral plaque caused by diabetes are likely to shift the ability of DPSCs to select the proliferative clones towards a main tendency to differentiate, therefore exhausting the original multipotent and stromal stem cell pool. From a clinical point of view, this effect would result in a deregulated ossification and in the inability of patients to exploit their own stem cell pool to trigger regeneration upon pathological conditions. Accordingly, DPSCs represent the responsible population for periodontal homeostasis, able to actively respond to different damages, by enhancing the repairing process [15]. Notably, periodontal therapy (especially the intensive kind) in diabetic patients in combination with glycaemia control is able to successfully restore a suitable periodontal microenvironment [33, 37, 38], therefore confirming the reversible epigenetic nature of T2D. Thus, it is also plausible that hyperglycaemia consequent to insulin resistance (the hallmark of T2D patients) may represent the key stimulus to change both the composition of the oral plaque and the biological features of DPSCs.

## 5. Conclusions

Our study strengthens the involvement of DPSCs in T2D-mediated PD. The finding that oral plaque may represent the direct biological modality by which diabetes impairs the dental pulp stem and progenitor cell pool could be of clinical significance.

## Figures and Tables

**Table 1:** Clinical features of patients.

	Age	Sex	Smoker
*Donor DPSCs*			
1	16	M	No
*Donor oral plaque*			
T2D-B	52	M	No
T2D-C	91	F	No
T2D-D	79	M	No
T2D-E	94	F	No
T2D-F	94	M	No
Ctl-G	22	M	No
Ctl-H	53	M	No
Ctl-I	82	F	No

**Table 2:** Multivariable linear regression analysis for the association of the normalized CFU yield (CFU/mg) and diabetes, adjusted for age.

Independent variable	Regression coefficient	*p*
T2D	-1.74	0.01
Age	-0.006	0.54

## Data Availability

The data used to support the findings of this study are available from the corresponding author upon request.
